# Mapping global evidence on injuries/trauma due to sexual and gender-based violence for research prioritisation and development of guidelines to mitigate their impact: a scoping review protocol

**DOI:** 10.1186/s13643-023-02345-8

**Published:** 2023-09-20

**Authors:** Desmond Kuupiel, Monsurat A. Lateef, Julian D. Pillay, Gugu G. Mchunu

**Affiliations:** 1 Faculty of Health Sciences, Durban University of Technology, Durban, 4001 South Africa; 2 Department of Public Health Medicine, School of Nursing and Public Health, University of KwaZulu-Natal, Durban, 4001 South Africa

**Keywords:** Gender-based violence, Sexual violence, Injuries, Practice guidelines

## Abstract

**Background:**

The World Health Organization recognises injuries as a growing global public health problem. While there are several causes of injuries and trauma, relevant research is mostly centred on road traffic accidents, burns, and drowning with less focus on violence-related injuries/trauma such as sexual and gender-based violence (SGBV). To identify priority research topics, prioritisation, and development of practice guidelines to mitigate the impact of injuries/trauma resulting from SGBV, this systematic scoping review will aim to map and describe the range of research relating to injuries/trauma due to SGBV in the global context.

**Methods:**

A scoping review guided by Arksey and O’Malley’s methodological framework will be conducted. Literature relating to injuries/trauma and SGBV will be searched in PubMed, SCOPUS, and PsycINFO, CINAHL, Web of Science, Google Scholar, Trip, guideline repositories, websites, and reference list of included articles. This study will include evidence sources focused on the epidemiological burden, guidelines for out-of-hospital and in-hospital care of victims, barriers or facilitators to reporting and obtaining healthcare, and approaches for mitigating injuries/trauma due to SGBV. The search will be limited to publications within 10 years (2012 to 2023). Two authors will apply the eligibility criteria to identify potentially relevant citations. The data will be extracted in duplicate and methodological quality assessed using varied tools (Mixed Method Quality Appraisal Tool; and Appraisal of Guidelines, Research and Evaluation instrument). The study will be reported in keeping with the Preferred reporting items for systematic reviews and meta-analyses extension for scoping reviews.

**Discussion:**

The scoping review will highlight existing literature on injuries/trauma due to SGBV and identify gaps to facilitate research prioritisation, development of guidelines, and resource allocation to alleviate the impact of injuries/trauma resulting from SGBV. This study’s findings will be disseminated via a series of meetings with key stakeholders (local and international) in the field of healthcare, policy, social welfare, GBV interest groups, and others. Also, the final scoping review results will be presented at relevant workshops, meetings, and conferences. Moreover, this study’s findings will be disseminated via journal publications and policy briefs.

**Supplementary Information:**

The online version contains supplementary material available at 10.1186/s13643-023-02345-8.

## Background

The World Health Organization (WHO) reports that injuries are a growing global public health problem [1]. In 2021, the WHO estimated that unintentional and violence-related injuries kill more than 4 million people around the world each year and constitute nearly 8% of all deaths as well as responsible for an estimated 10% of all years lived with disability each year [1]. Deaths due to unintentional and violence-related injuries are about 32% more than the number of fatalities that result from malaria, tuberculosis, and HIV/AIDS combined, yet much attention is seemingly given to malaria, tuberculosis, and HIV/AIDS compared to injuries [2]. Injuries result from road traffic accidents, falls, drowning, burns, poisoning, and acts of violence against others such as gender-based violence (GBV) among other causes [1, 3]. However, GBV is one of the neglected causes of injuries that is silently affecting the lives of many people, especially women [4, 5].

Gender-based violence also referred to as sexual and gender-based violence (SGBV), is any damaging act of sexual, physical, psychological, mental, or emotional abuse that is performed against a person’s will, and is based on socially attributed (i.e. gender) disparities between males and females [6–9]. Evidence shows that one out of every three women aged 15 to 49 will be subjected to physical or sexual assault in lifetime [10–12]. The prevalence of lifetime intimate partner violence estimates range from 20% in the Western Pacific, 22% in high-income countries and Europe, and 25% in the WHO Regions of the Americas, to 33% in the WHO African region, 31% in the WHO Eastern Mediterranean region, and 33% in the WHO South-East Asia region [8]. Women, girls, and boys are vulnerable populations of SGBV [9]. In times of conflict, SGBV rises sharply [13]. For instance, rape is used as a tactic of war and terror to humiliate, dominate, or undermine social relationships and ethnic identity in women’s bodies [13]. Sexual gender-based violence survivors are usually left to fend for themselves when support networks and local services fall apart, and facilities are damaged or destroyed [13].

The consequences of SGBV may include serious short- and long-term effects such as physical, mental, sexual, and reproductive health problems for women survivors and their communities [14–16]. Physical injuries, unexpected pregnancies, fistulas, sexually transmitted illnesses like HIV, and death are all possible outcomes of such mindless violence [9, 17, 18]. In addition, survivors are frequently subjected to social rejection, which makes them more vulnerable to further abuse and exploitation [8]. As a result, the health sector has an important role to play in preventing and responding to violence, particularly against women through a multi-sectoral approach [8].

The WHO advocates for countries to generate evidence on what works and on the magnitude of the problem by carrying out population-based surveys or including violence against women in population-based demographic and health surveys [8]. The WHO further advocates the inclusion of violence against women in countries’ surveillance and health information systems [8]. To this end, knowledge of existing research on injuries/trauma due to SGBV is essential to enable to identify literature gaps, prioritise research, and develop guidelines to mitigate their impact. This scoping review, therefore, seeks to systematically map evidence and describe the range of research on injuries due to SGBV within the last decade in the global context, since no such study currently exists in the literature. The study would form a basis for initiating further studies to address specific gaps in this area of research, with particular emphasis on reaching towards achieving the Sustainable Development Goal targets linked to injury, universal health coverage, violence prevention, mental health, and substance use [19].

## Methods

To achieve this scoping review objective, the Arksey and O’Malley methodological framework [20] will be employed to scope literature relating to injuries/trauma and SGBV in the global context. Identifying the research question. identifying relevant studies, study selection, data charting and collation, and summarising and reporting the results [21, 22] are the steps this study will follow, as per the framework to be applied.

### Identifying the research question

This scoping review question will be what research evidence on injuries/trauma due to sexual and gender-based violence within the last decade exists in the global context? The Population, Concept, and Context framework [23] used to illustrate the appropriateness of this scoping review question is presented as part of the study eligibility criteria (Table 1). The sub-questions for this scoping review will be as follows:

1. What evidence on the epidemiological burden of injuries/trauma due to SGBV in the global context over the last decade exists?
2. What guidelines are available or absent for the management of SGBV victims with injuries/trauma (e.g., genital injuries and psychological trauma) in the hospital and out-of-hospital settings?
3. What are the reported barriers and facilitators to reporting and obtaining healthcare by victims of SGBV with injuries/trauma?
4. What evidence on approaches used to mitigate injuries/trauma due to SGBV exist?

**Table 1:** Study eligibility criteria

Eligibility criteria	Inclusion criteria	Exclusion criteria
Population	Males and females of all ages with injuries/trauma linked to SGBV	
Concept	Injuries/trauma linked to SGBV: This will include both physical (fatal and non-fatal) injuries and psychological/mental trauma resulting from intimate partner violence, rapid and other forms of sexual assault	Injuries linked to consensual sex
Context	• Epidemiological burden • Guidelines for out-of-hospital or in-hospital care of victims • Barriers and facilitators to reporting and obtaining healthcare • Approaches for mitigating injuries/trauma due to SGBV	
Setting	Global	
Study designs/publication type	• Original research employing primary study designs such as quantitative, qualitative, or mix-methods • Systematic reviews and /or meta-analysis • Guidelines such as practice guidelines for clinical care or strategy for mitigating the impact of trauma due SGBV • Peer reviewed papers	• Reviews such as literature review, rapid review, and expert review • Abstracts only • Conference documents • Editorials • Grey literature such reports, theses and dissertations
Language	All publication languages	
Time frame	Publications between 2012 and 2022	

### Literature searches

The search will aim to identify relevant peer-reviewed papers to answer the review questions. To achieve this, we will conduct a comprehensive search in the following electronic databases from January 2012 to the search date in 2023: PubMed/MEDLINE, SCOPUS, CINAHL, PsychINFO, and guideline clearinghouses (Scottish Intercollegiate Guidelines Network, Trip, and Guidelines International Network). We will further search the WHO website, relevant Ministries, Departments, and Governmental websites as well as UNFPA website for relevant literature. Google Scholar search engine will also be used to search for relevant literature. A search strategy developed in consultation with an information scientist will be employed for database literature searches. The search string will involve a combination of keywords (“gender-based violence”, “gender based”, “violence”, “gender”, “based”, “sex offences”, “sexual”, “sexual violence”, “injuried”, “injuries”, “wounds and injuries”, “wounds”, “injurious”, “injury s”, “injuryed”, “injurys”, “injury”, “trauma”), Boolean operators (AND/OR), and Medical Subject Heading (MeSH) term (Please see Table 2 for a pilot search strategy in PubMed). Due to the variability of the databases, the syntax will be modified accordingly. Similarly, the information scientist will be involved in the website literature searches. Aside from the database and website searches, we will manually explore the reference list of the included evidence sources for additional relevant literature. Search filters such as language and publication type will not be applied; however, the search date will be limited to publications within 10 years (from 2012 to 2023). PRISMA-S extension for reporting literature searches will be employed to document the searches [24]. All search yields will be imported onto an EndNote Library X20 for citation management.

**Table 2:** Pilot search strategy in PubMed

Date	Database	Query	Yield
23/08/2023	PubMed	**#1:** Search: injury OR trauma "injurie"[All Fields] OR "injuried"[All Fields] OR "injuries"[MeSH Subheading] OR "injuries"[All Fields] OR "wounds and injuries"[MeSH Terms] OR ("wounds"[All Fields] AND "injuries"[All Fields]) OR "wounds and injuries"[All Fields] OR "injurious"[All Fields] OR "injury"[All Fields] OR "trauma"[MeSH Subheading] OR "trauma"[All Fields] OR "traumas"[All Fields])	1,944,443
		**#2:** Search: sexual violence OR gender-based violence OR sexual assault "sex offenses"[MeSH Terms] OR ("sex"[All Fields] AND "offenses"[All Fields]) OR "sex offenses"[All Fields] OR ("sexual"[All Fields] AND "violence"[All Fields]) OR "sexual violence"[All Fields] OR ("gender based violence"[MeSH Terms] OR ("gender based"[All Fields] AND "violence"[All Fields]) OR "gender based violence"[All Fields] OR ("gender"[All Fields] AND "based"[All Fields] AND "violence"[All Fields]) OR "gender based violence"[All Fields]) OR (("sexual behavior"[MeSH Terms] OR ("sexual"[All Fields] AND "behavior"[All Fields]) OR "sexual behavior"[All Fields] OR "sexual"[All Fields] OR "sexually"[All Fields] OR "sexualities"[All Fields] OR "sexuality"[MeSH Terms] OR "sexuality"[All Fields] OR "sexualization"[All Fields] OR "sexualize"[All Fields] OR "sexualized"[All Fields] OR "sexualizing"[All Fields] OR "sexuals"[All Fields]) AND "assult"[All Fields])	45,135
		**#3:** Search: (injury OR trauma) AND (sexual violence OR gender-based violence OR sexual assault)	8,851
		**#4:** Filters applied: from 2012/1/1—2023/8/23	5,354

### Articles selection process

**Fig. 1:**
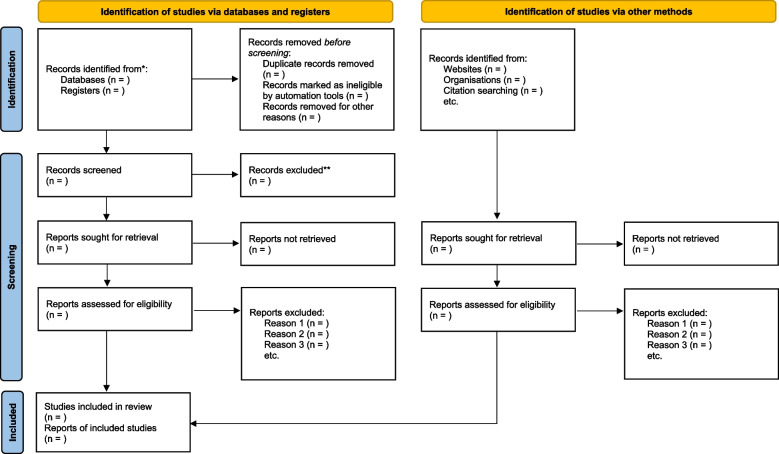
PRISMA 2020 flow diagram

This study selection tool will be developed a priori in Google Forms using the items in the inclusion criteria (Table 1) and pilot-tested. Subsequently, the EndNote library will be de-duplicated using the “Find Duplicate” function. During the title and abstract, and full-text screening phases, two authors (DK and ML) will independently sort the articles into two groups—“include” and “exclude” guided by this study’s eligibility criteria using the pilot tested form. For articles without abstracts, only the full text will be assessed. A third author (GM or JD) will resolve all discrepancies (non-consensus) resulting from the study selection between DK and ML. Efforts will be made to obtain full-text articles that are not published open access by employing the services of the Durban University of Technology Library Services. If not obtainable in this way, we will additionally make a formal request to the original authors via email and ResearchGate to obtain full-text articles for screening. The 2020 PRISMA flow diagram will be used to document the article selection process (Fig. 1).

### Quality appraisal

To assess the risk of bias and describe the quality of the included studies, the Mixed Method Quality Appraisal Tool (MMAT) Version 2018 [25] will be used to assess the methodological quality of all included articles that employed a primary study design. The MMAT has sections for quantitative, qualitative, and mixed-methods study designs; hence, this study’s choice to employ it for the appraisal. The appropriateness of the study’s objective, suitability of the study design, participant recruitment, data collection, data analysis, and result/findings presented will be appraised. We will grade the quality of studies using a quality score ranging from 50% as low quality, 51–75% as average quality, and 76–100% as high quality. To better inform the quality of the included studies, a detailed presentation of the ratings of each criterion will be additionally provided. Though quality appraisal is not essential, this step is essential to further enable us to identify relevant research gaps. However, to describe the quality of the existing guidelines for managing SGBV victims with injuries/trauma in either in-hospital or out-of-hospital settings, we will employ the international Appraisal of Guidelines, Research and Evaluation (AGREE II) tool to evaluate the rigour of the development of all included guidelines [26]. The included guidelines will be graded as low quality (AGREE domain 3 scores < 60%) and high quality (an AGREE domain 3 score ≥ 60%). The quality appraisal will be performed by two authors independently and any disagreement addressed by a third author.

#### Charting the data

For data abstraction, a spreadsheet will be created and pilot-tested with 10% of the included evidence sources. To ensure that the form captures all relevant data to answer the review question, the necessary changes will be made. Following a thorough reading of the full texts, two reviewers will independently extract all relevant data from the included evidence sources. The data from the included evidence sources will be extracted using a hybrid approach that combines inductive and deductive reasoning [27]. Due to the differences between the design of primary studies and guidelines, two separate data abstraction forms will be employed. For the included primary studies, the study characteristics, i.e. author(s), publication year, study title, study aim/objective, and study methods such as geographical location (country), study design, study population, characteristics of the study sample, and analysis approach will be extracted. We will also extract the results/findings reported by the included studies such as injury type/trauma, incidence, prevalence, mortality, reported barriers and facilitators to reporting and obtaining healthcare, and approaches for mitigating injuries/trauma due to SGBV. For each of the included guidelines, the following information will be extracted:

#### Guideline characteristics

- Developer (government department, professional body/society, and others)
- Date/date last updated
- Publisher
- Country where the guideline was developed

#### Guideline scope and purpose

- Guideline title
- Guideline aim/objective
- Guideline question(s)
- Targeted population
- Topic/type of injury/trauma
- Targeted user
- Recommendation(s)

#### Guideline quality

- Development/adolopment approach (de novo or alternative/adaptive methods).
- Evidence grading system or tool
- Rigour of development (AGREE-II Domain 3 components)
- Strength of recommendations

### Collating, summarising, and reporting the results

A descriptive analysis will be conducted to describe the themes (topics) aligned with this scoping review’s question based on initial coding and categorisation [27]. Relevant topics relating to injuries/trauma and SGBV will be identified and described. This review report will focus on four thematic areas: selection of the evidence sources, characteristics of the evidence sources, and study results/findings (epidemiological burden of injuries due to SGBV; availability, scope, and purpose of the guidelines; barriers to healthcare by victims with injury/trauma due SGBV); and the quality of the studies/guidelines. Other relevant emerging topics essential to answering this scoping review’s questions will also be described appropriately in line with the thematic areas. A summary of the findings for each theme will be reported using qualitative description, but tables, figures, and maps where appropriate. STATA version 14.0 will be used to perform all descriptive analyses. We will further describe areas where evidence is lacking and make recommendations for further research. This study will be reported in keeping with the Preferred Reporting Items for Systematic Reviews and meta-analyses extension for scoping reviews (PRISMA-ScR) checklist [28].

## Discussion

This systematic scoping review aims to map and describe the range of research relating to injuries/trauma due to SGBV in the global context in order to identify priority research topics, prioritise, and develop practice guidelines to mitigate the impact of SGBV injuries/trauma. Injuries are a growing global public health problem. Injuries occur due to a variety of causes; however, research on injuries/trauma is primarily focused on traffic accidents, burns, and drowning, with less emphasis on violence-related injuries/trauma such as sexual and gender-based violence (SGBV), hence informing this study. This scoping review will be limited to research evidence within the last decade. Focusing on more recent publications will allow the study to capture the most up-to-date information and research findings and ensure that the review is relevant and reflects current knowledge, practices, and what interventions might be needed. It is hoped that this study will highlight existing literature on SGBV injuries/trauma and identify gaps to facilitate research prioritisation, development of guidelines, and resource allocation to reduce the impact of SGBV injuries/trauma.

This study’s findings will be disseminated to the scientific community through traditional means such as publications in relevant peer-reviewed journals and presentations at relevant conferences. Findings will be shared with local and international policymakers and practitioners through targeted stakeholder meetings or workshops, policy briefs, and a webinar. The findings will also be shared with the general public or the non-scientific community through infograms, online, blogs, print media, and radio, and webinars.

### Supplementary Information

**Additional file 1:** PRISMA-P Checklist.

## Data Availability

All data sources will be presented in the form of references.
